# The Holy month of Ramadan: A blessed union (Etihad) of societal collective effervescence, belonging and positive mental health for all

**DOI:** 10.12688/f1000research.162960.1

**Published:** 2025-03-25

**Authors:** Richard Mottershead, Muhammad Arsyad Subu, Mustafa Habeb, Wegdan Bani-Issa, Jacqueline Maria Dias, Mini Abraham, Vidya Seshan, Alounoud Almarzooqi, Maryam Butti Saeed Alshamsi, Muna Al-Tamimi, Janisha Kavumpurath, Sadeq AL-Fayyadh, John Hall, Ghada Shahrour, Sarah Sanad, Sindya Saif Almarzrouei, Sultan Abdullah Mohammed Alshaer, Abdulla Yoqoub AlHammadi, Mohammed Baqer Habeeb Abd Ali

**Affiliations:** 1 Nursing Department, College of Health Sciences, University of Sharjah, United Arab Emirates, University of Sharjah, Sharjah, United Arab Emirates; 2 Faculty of Nursing and Midwifery Universitas Binawan Jakarta, Indonesia, Universitas Binawan, East Jakarta, Special Capital Region of Jakarta, Indonesia; 3 Department of Health Care Management, College of Health Sciences, University of Sharjah, Sharjah, Sharjah, United Arab Emirates, University of Sharjah, Sharjah, Sharjah, United Arab Emirates; 4 College of Nursing, University of Baghdad, Baghdad, Iraq, University of Baghdad, Baghdad, Baghdad Governorate, Iraq; 5 SEHA, Sakina, Abu Dhabi Health Services Company, Abu Dhabi, United Arab Emirates, SEHA, Abu Dhabi, United Arab Emirates; 6 College of Nursing, RAK Medical and Health Sciences University, Ras Al Khaimah, United Arab Emirates, Ras Al Khaimah Medical and Health Sciences University, Ras Al Khaimah, United Arab Emirates; 7 Dubai Medical University, College of Nursing, Dubai, UAE, Dubai Medical University, Dubai, United Arab Emirates

**Keywords:** Ramadan, Societal Collective Effervescence, Belonging, Tolerance, Community, Mindfulness, Reflection, Mental Health

## Abstract

Ramadan is a month-long religious festival and observed globally by Muslims, characterised by intermittent fasting, prayer, reflection and a focus on community. Fasting is one of the five pillars of Islam and has well documented positive impacts on physical health. An article by Bandarian et al., (2021) highlighted the research gaps in Ramadan fasting studies relating to health and disease. The article called for more research studies on different health and disease conditions other than nutrition and metabolic disorders. The authors on this correspondence article continue this call and respectfully articulate the wider societal impact and benefits of Ramadan through its positive impact, through embracing multicultural, multinational and multifaith communities within the United Arab Emirates.

## Introductions

Islam provides a beacon of light and hope for the collective cohesion of societies within the Arab world and communities around the World. As with all religions, the path of enlightenment can have distractors but the purity of Islam within the fertile soil of the Abrahamic family of religions, celebrates and promotes peace above all. The holy month of Ramadan embraces and encourages all to participate in the practice of mindfulness. For those devoted to the Islamic faith this is through pray and fasting. Yet, for visitors to the United Arab Emirates (UAE) and other Arab nations, Ramadan creates opportunities for community gatherings for all faiths, denominations, and nationalities. Iftar (the breaking of the fast) is enjoyed in many communities which aids in promoting a sense of belonging within Islamic communities, but as importantly within the multicultural communities within the UAE. This practice and policy empower visitors to invariably become interwoven into the shared identity, history and culture of the UAE and the wider Arab World, through the peaceful embrace of Islam. This ethos of ‘Etihad’ (Union/Unity) has been a source of substance, prosperity and identity shaping the Emirates.

This letter to the Editor seeks to present Ramadan as a pilgrimage that can be taken by all who may wish to enhance mental well-being and to better understand the societal benefits, enriching multicultural communities due to Etihad through Ramadan within the UAE.

## Societal collective effervescence: Community solidarity through Ramadan

Durkheim (1965) sought to create an awareness through the framework of structural functionalism which seeks to build theory through the actualisation that society is a complex system. Durkheim (1965) was concerned with the concept of how certain societies maintain internal stability and survive over time. His proposition that societies tend to be segmented, with equivalent parts held together by shared values, common symbols or systems of exchanges is of interest to article as Ramadan is seen a shared experience positively affecting perceptions and experiences for non-Muslims. Durkheim (1965) and Goffman (1961) sought to investigate this solidarity through social bonds, based on common sentiments and shared moral values that were recognised to be strong among those exposed to ‘total institutions’. Within this seminal work, ‘total institutions’ were conceived to be institutions where a great number of people, in similar situations were detached from a sider community for a time period. The authors suggest that this concept could be applied to the ‘immigrant/expat’ communities that may be non-Muslim but reside within countries such as the UAE. Reflectively, the authors propose that Durkheim’s (1965) argument that complex societies are held together by organic solidarity, i.e., social bonds, based on specialisation and interdependence could be extended to Ramadan which appears to support a sense of belonging to those who wish to participate.

Evidence from the authors own life stories living within the UAE illuminates a reported congruence between Emirati’s, residents, and visitors of this nation. The authors (both Muslim and non-Muslim) believe that the strength of Ramadan in terms of belonging, has strong links to the sociologist theory of Durkheim (1915, 1995) who described a process entitled ‘collective effervescence’. This concept was used by Durkheim within his research on religion and refers to collective effervescence as moments within societal life when a group of individuals come together in order to perform a religious ritual ( Durkheim, 1995). During this ritual, the group communicates holistically in the same thought and participates in the same action, which serves to unify a group of individuals. Durkheim observed that when individuals come into close contact with one another and assemble in collective purpose, a certain “electricity” is created and released, leading participants to a high degree of collective emotional excitement (1995). Whilst Durkheim’s research did not focus on Islam or Ramadan the authors suggest that this concept highlights the impersonal, extra-individual force, which is a core element of Ramadan as transporting the individual into a new, ideal realm, elevating them both internally and externally from themselves, and making them feel as if they are in contact with an extraordinary energy. It is believed by the authors that this same concept relates to the unifying properties present within the establishment of belonging and Ramadan as Muslims and non-Muslims are brought together and given a shared experience within a reflective and mindful practice of fasting. Studies attest to the positive mental health experienced by those participating in Ramadan. Sulaiman et al. (2023) who noted reductions in depression and anxiety levels during Ramadan of a sample of 639 adults who participated in Ramadan. Likewise, this was mirrored within a study in Saudi Arabia that noted a reduction in depression, anxiety, and stress within sub-groups engaging in fasting, reflection and a focus on community ( Gosadi, 2025). Briki, Aloui, and Bragazzi (2019) stipulate that the Fasting ritual of Ramadan can act as a buffer against intense emotions, reducing both positive and negative effects, and lowering levels of depression. Similarly, Qureshi (2011) articulates how Ramadan can reshape of an individual’s character through infusing a sense of consciousness and piety. These findings link into the authors beliefs that Ramadan can be an effective therapeutic process to promote mental health and enhance a sense of belonging for all.

## Welcoming and fostering a sense of belonging

Within a multinational community such as in the UAE, communities sharing the joys of Ramadan can create a new sense of purpose, developing a sense of worth and meaningfulness greater than themselves in which to galvanise aspirations through ‘Etihad’ to join a greater and welcoming community as exists within the UAE. Goodenow (1993) supports this view in that shared experiences can seek to establish a sense of belonging which in turn increases wider success and motivation. Mottershead (2022) argued that creating belonging and support were especially important for motivation, engagement and performance of those coming from past lives who wish to better themselves. Indeed, this is a major attraction to the UAE for so many visitors seeking better lives. This point is supported by Becker and Luthar (2002) who assert that one of the main features affecting those having experiences economically disadvantages was an absence of belonging to those former groups. The authors argue that groups could be extended to former home countries of visitors to the UAE. For visitors to the UAE who may have come hoping for new beginnings through migration, Ramadan offers a shared experience through peaceful blessings of Islam.

In conclusion, the ethos of Ramadan within the multicultural, multinational society of the UAE is to create an authentic warm welcome to promote inclusion to visitors. This practice is in no small way a co-incidence. It is based in the time-honoured Arab traditions, historically stemming to nurture travellers who pass-by on their journeys. Emirati hospitality honours the ‘guest’ and seeks to ensure they feel welcomed and thereby ridding them of feeling out of place and providing a home away from their home. This most sacred of caveats is the beating heart of the Holy month of Ramadan and is interwoven into Hadiths (sayings of the Prophet Muhammad, peace be upon him) that emphasize generosity and treating guests with kindness. This generosity has been experienced by generations of visitors to the UAE, and we (the authors) hope that many more generations experience this blessing.

## Ethics and consent

Ethics and consent were not required.

## Author contributions

RM conceptualization, all authors writing – original draft preparation, review and editing.

## Data Availability

No data are associated with this article.
