# Supplementary material for: The Holy month of Ramadan: A blessed union (Etihad) of societal collective effervescence, belonging and positive mental health for all
Source: F1000Res. 2025 Mar 25;14:327. [Version 1] doi: 10.12688/f1000research.162960.1 (PMC12144488; doi:10.12688/f1000research.162960.1)
Supplement: Peer review history

# Reviewer report: Reviewer response for version 1

**DOI:** 10.5256/f1000research.179246.r388210

**Published:** 2025-06-06

**Authors:** Sayid Ridho

**Affiliations:** 1 Syarif Hidayatullah State Islamic University, Ciputat, Indonesia

**My Review Report for this article:**

 1.Is the rationale for commenting on the previous publication    clearly described?

  answer: **Yes**

 2. Are any opinions stated well-argued, clear and cogent?

 answer: **Partly**

 The mechanisms of how Ramadan creates these effects for all communities ( social collective effervescence, belonging and positive mental health) aren't described with absolute clarity.

 3.Are arguments sufficiently supported by evidence from the published literature or by new data and results?

 answer: **Partly**

 The arguments regarding how Ramadan concretely fosters inclusion and "collective effervescence" for non-Muslims still require more specific observational evidence or data.

 4. Is the conclusion balanced and justified on the basis of the presented arguments?

 answer: **Partly**

 There are found a lack of balance with an acknowledgment of challenges or limitations

**Summary:**

 To elevate academic sound and objectivity, the theoretical and concepts should be applied precisely using sociological concepts, supported by more detailed evidence and ensuring all cited studies directly support the arguments properly. Importantly, clarify the basic mechanisms/relationship through which Ramadan can impact in human body (transforming it academically) to social collective effervescence, belonging and positive mental health by make a scheme or pathway. Finally, in the conclusion, rather than just summarizing, to acknowledge the complexities, it will be better to add the synthesis of the key arguments to offer broad implications or future research directions.

Are arguments sufficiently supported by evidence from the published literature or by new data and results?

Partly

Is the conclusion balanced and justified on the basis of the presented arguments?

Partly

Is the rationale for commenting on the previous publication clearly described?

Yes

Are any opinions stated well-argued, clear and cogent?

Partly

Reviewer Expertise:

Islamic Medicine; Internal Medicine; Stem Cell

I confirm that I have read this submission and believe that I have an appropriate level of expertise to confirm that it is of an acceptable scientific standard.

# Reviewer report: Reviewer response for version 1

**DOI:** 10.5256/f1000research.179246.r374997

**Published:** 2025-04-14

**Authors:** Shwikar Mahmoud Etman Othman

**Affiliations:** 1 Faculty of Health and Medical Sciences, Adelaide Medical School, University of Adelaide, North Terrace, Australia; 2 School of Nursing, Curtin University, perth, Australia

Please find the attached word document detailing my feedback.- *https://f1000research.s3.amazonaws.com/linked/721400.Reviewer_comments_13_4_2025.docx*

Are arguments sufficiently supported by evidence from the published literature or by new data and results?

Yes

Is the conclusion balanced and justified on the basis of the presented arguments?

Yes

Is the rationale for commenting on the previous publication clearly described?

Yes

Are any opinions stated well-argued, clear and cogent?

Yes

Reviewer Expertise:

NA

I confirm that I have read this submission and believe that I have an appropriate level of expertise to confirm that it is of an acceptable scientific standard.
